# Annual Meetings of Hospitals

**Published:** 1920-03-27

**Authors:** 


### Annual Meetings of Hospitals.

### The National Hospital for the Paralysed and Epileptic.

Sir Frederick Macmillan, the Chairman of the Board of Management, took the chair at the annual meeting on Tuesday, March 23, and made the important announcement that the whole of the medical wards of the hospital would be closed on December 31 if by the end of July sufficient support had not been promised to maintain the hospital free of debt for the next three years. The expenditure has risen from £15,932 in 1913 to £33,675 in 1919, and the cost per occupied bed from £1 14s. 8d. to £3 3s. 11d. in the same period. In the former year the Board, in the annual report, thought it necessary to apologise for the cost being so high, owing to the increase in the cost of drugs, etc. !

An appeal for a Special Maintenance Fund is being issued, and the rich man is asked to promise £1,000 this year and £500 in the two succeeding years, or at least £500 now and £250 in 1921 and 1922. The man of moderate means, it is hoped, may give £100 and two sums of £50, while those who are less well off are asked to provide £10 and £5 to come.

The deficit last year was nearly £7,000, but the financial position has not been caused by want of prudence, as it has not been the inclination of the Executive to undertake large enterprises without the means to carry them out.

### Samaritan Free Hospital.

At the annual Court of Governors held last week it was pointed out that the income of £9,244 shows a considerable decrease on that of 1918, when it was £9,996, while the expenditure has gone up from £10,052 to £11,795, the result being that 1919 ended with a deficit of £2,550. The position, the committee emphasises, is a very serious one. During the period under review 1,069 in-patients have been admitted, the number being the highest since the foundation of the institution. Nearly 1,000 more new out-patients received attention last year than in 1918, the increase in the total of the attendances being over 2,000. The experiment, started in 1916, of having a convalescent home for the sole use of the hospital having proved such a great success the committee has purchased a modern house, the freehold of which cost £1,500, and converted it into a home more suitable for the requirements of the patients than the original ancient cottage.

### Great Northern Central Hospital.

The Great Northern Central Hospital, Holloway Road, the annual meeting of which was held last week, treated 2,503 in-patients in 1919. The number was smaller than in the two previous years, owing to the closing of several of the wards during an extended period for structural repairs and cleaning, and also in consequence of an outbreak of infection in the children's ward. A further large increase in the number of out-patients, however, was recorded, 29,276, whose attendances numbered 173,637, having been cared for, as compared with 24,320 and 124,487 attendances in 1918. Over 1,110 cases of venereal disease, involving 7,146 attendances, were dealt with last year. The income of £50,909 was lower by about £100 than that in 1918, and ordinary expenditure increased from £49,002 in that year to £52,431 during the following twelve months. It is hoped that detailed plans of the scheme for the extension of the hospital, which will form the official War Memorial of the Borough of Islington, will be received by May. Sir Aston Webb has been appointed to assess competitive designs to be submitted by four selected candidates. The names of all Islingtonians who fell in the war will be inscribed in the new public entrance hall, which will form part of the extension scheme. Pay wards for persons of limited incomes have been in existence at the institution for many years, and out-patients are now invited to contribute a stated sum towards the cost of their medicine and dressings. The title of the hospital is to be altered to the "Royal Northern Hospital."

### Hospital for Women, Soho.

In the absence of the Earl of Shaftesbury, Earl Winterton, M.P., presided last week over the Annual Court of Governors of the Hospital for Women, Soho Square, which last year admitted 1,073 in-patients, as compared with 1,020 in 1918. The pressure on available space has been exceptional, and emergency beds have had to be set up on several occasions. Out-patients in 1919 numbered 4,292, with an aggregate of 14,056 attendances, as against 3,760 and 12,106 attendances during the preceding twelve months. The balance sheet for the period under review showed a deficit of £2,170, due, in the first place, to the fact that the legacies available for general purposes received amounted to only £225, and secondly to increased expenditure, which reached the record sum of £10,736. During the past year the permanent endowment fund of the hospital has been increased by £2,392. The mortgage debt has been reduced by £250 through the generosity of the King's Fund, and now stands at £1,750. It costs the institution £87 a year in interest, and the Committee earnestly appeals for contributions towards its reduction. Sir William F. Clerke, Bart. (Deputy Chairman of the Committee), has been nominated to fill the office of treasurer of the hospital, rendered vacant by the resignation and subsequent death of Mr. F. A. Bevan, who had held the position for upwards of twenty-nine years.

### London Lock Hospital.

Presiding at the annual meeting of the London Lock Hospital last week, Mr. J. F. W. Deacon, Vice-President and Deputy-Chairman of the Board of Management, stated that the audited accounts showed a deficit on the Rescue Home revenue account of £1,593, and pointed out that after allowing for the grant in aid received from the L.C.C., a sum of probably £10,000 would be required this year to maintain the charity. At the urgent request of the King's Fund the Board was about to issue an appeal for £80,000 for building a new wing to the existing female hospital in Harrow Road, to be devoted to the treatment of expectant mothers and their infants. The new wing, when completed, would provide accommodation for women, children, and babies suffering from venereal disease, and would meet the demand for treatment of a class that was practically unprovided for at the present time, and whose claims constituted a unique call upon the benevolence of the charitable public. The Board hoped to raise the sum required by the end of this year, in order that they could commence building. In the meantime it was of vital importance that the public should provide the £14,000 required for general maintenance. Out-patients at the male hospital had increased from 3,367 in 1918 to 5,401 last year, while their number of attendances had gone up by over 50 per cent. As an instance of how the institution is regarded by the profession, the Committee states that 276 practitioners from all parts of the world have attended its practice.

